# High temperature ferromagnetism in π-conjugated two-dimensional metal–organic frameworks†
†Electronic supplementary information (ESI) available: (1) Computational methods; (2) electronic band structures of NiMPc MOF monolayers (Fig. S1); (3) calculation results for NiMnPc MOF monolayers with NiO_4_ and NiS_4_ moieties (Fig. S2); (4) ferromagnetic transition temperatures of 2D Heisenberg model with single-ion anisotropy (Fig. S3); (5) structure and energetics of bulk NiMnPc (Fig. S4 and S5); (6) atomistic coordinate data of NiMPc MOF monolayers. See DOI: 10.1039/c6sc05080h



**DOI:** 10.1039/c6sc05080h

**Published:** 2017-02-08

**Authors:** Wenbin Li, Lei Sun, Jingshan Qi, Pablo Jarillo-Herrero, Mircea Dincă, Ju Li

**Affiliations:** a Research Laboratory of Electronics , Massachusetts Institute of Technology , Cambridge , Massachusetts 02139 , USA; b Department of Chemistry , Massachusetts Institute of Technology , Cambridge , Massachusetts 02139 , USA . Email: mdinca@mit.edu; c Department of Nuclear Science and Engineering , Department of Materials Science and Engineering , Massachusetts Institute of Technology , Cambridge , Massachusetts 02139 , USA . Email: liju@mit.edu; d Department of Physics , Massachusetts Institute of Technology , Cambridge , Massachusetts 02139 , USA

## Abstract

We use first-principles calculations to show that the square symmetry of two-dimensional (2D) metal–organic frameworks (MOFs) made from octaamino-substituted phthalocyanines and square planar Ni^2+^ ions, which enable strong conjugation of π electrons, has a critical impact on the magnetic properties of the lattice. In particular, we predict the unexpected emergence of a rare high-temperature ferromagnetic half-metallic ground state in one case. Among charge neutral MOFs made from (2,3,9,10,16,17,23,24)-octaiminophthalocyanine (OIPc) metallated with divalent first-row transition metal ions (M-OIPc; M = Cr^2+^, Mn^2+^, Fe^2+^, Co^2+^, Ni^2+^, Cu^2+^, Zn^2+^) and connected through square planar Ni-bisphenylenediimine moieties, NiMn-OIPc exhibits a half-metallic and ferromagnetic ground state with a large exchange energy resulting from the unique strong hybridization between the d/π orbitals of Mn, the Pc ring, and the Ni-bisphenylenediimine nodes. Notably, we show that for NiMn-OIPc there is a considerable difference between the ferromagnetic ordering temperature (*T*_c_) predicted by a 2D Ising model, which exceeds 600 K, and a *T*_c_ of 170 K predicted by our more realistic Monte Carlo simulation that includes magnetic anisotropy. Critically, our simulations adopt two spin models that incorporate magnetic anisotropy in the form of exchange anisotropy and single-ion anisotropy. We further show that in the bulk, 2D layers of NiMn-OIPc adopt a slipped-parallel stacking configuration, and exhibit interlayer magnetic coupling that is sensitive to the relative in-plane displacement between adjacent layers. These results highlight the critical role of magnetic anisotropy in modeling the properties of 2D magnetic systems. More generally, it demonstrates that strong hybridization between open-shell ions and delocalized aromatic π systems with appropriate symmetry, combined with large magnetic anisotropy, will be an effective design strategy to realize ferromagnetic 2D MOFs with high *T*_c_.

## Abstract 2

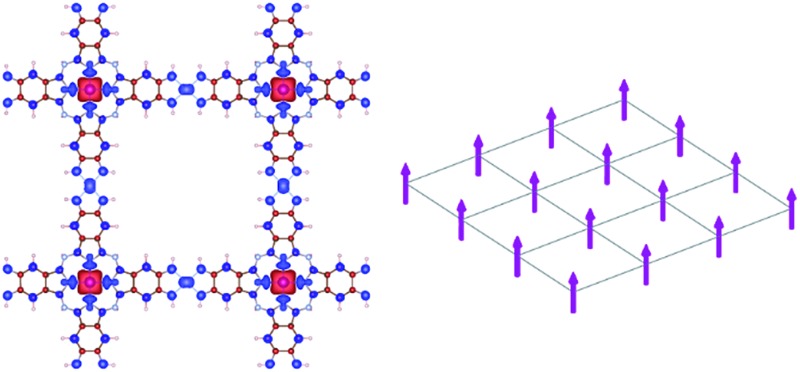
Simulations demonstrate the critical roles of π-conjugation and large magnetic anisotropy in realizing high-temperature ferromagnetic 2D metal–organic framework, which is also half-metallic.

## Introduction

A central focus of current research at the nanoscale is two-dimensional (2D) materials – planar sheets of materials with atomic thickness.^1^ 2D materials such as graphene and transition metal (TM) dichalcogenides exhibit fundamentally new properties that could be essential for next-generation electronic, photonic and renewable-energy applications.^2–6^ Recently, 2D metal–organic frameworks (MOFs)^7–16^ have emerged as a new class of hybrid inorganic–organic 2D materials marked by their intrinsic structural porosity, chemical tunability and high electrical conductivity.^17^ These materials are synthesized through a bottom-up approach, by combining aromatic organic moieties that can ligate square-planar metal ions. The delocalization of π electrons in the 2D MOFs leads to high electrical conductivity that is uncommon in conventional MOFs. This enables a wide range of new functionalities, including chemiresistive sensing^15,18^ and electrochemical oxygen reduction.^19^ Finally, the delocalized electronic states expected for these materials may give rise to exotic new physics including topological insulator behavior,^20,21^ the quantum anomalous Hall effect,^22^ and flat-band ferromagnetism.^23^

**Fig. 1:**
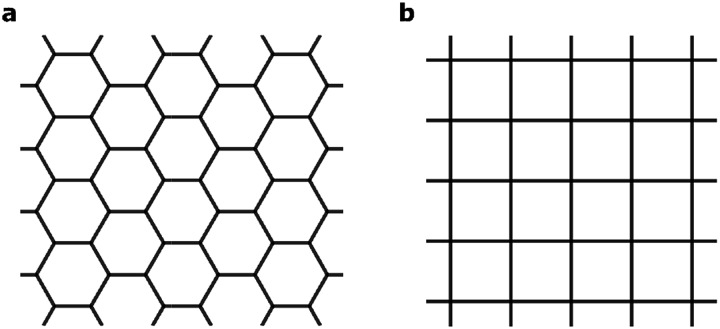
Schematics of (a) honeycomb and (b) square lattices.

The 2D MOFs synthesized so far^8–15^ are made from benzene- or triphenylene-derived ligands with imine, phenol, or thiophenol functionalities that bind a variety of late-transition-metal ions in a square planar environment. These 2D MOFs exhibit honeycomb lattices that stack in the third dimension, in a manner analogous to graphene and graphite. A schematic of such 2D honeycomb lattices, contrasted with 2D square lattices, is shown in Fig. 1. Although the presence of metals and the greater compositional variety of 2D MOFs allows significant modulation of band dispersion, absolute band energy, Fermi energy, and density of states, all 2D MOFs studied thus far have hexagonal symmetry and consequently exhibit electronic structures that mimic that of graphene symmetry-wise. Deviations from hexagonal symmetry should give rise to band structures that deviate entirely from those of hexagonal lattices, giving rise to electronic properties that diverge fundamentally from those expected from hexagonal 2D symmetry. It is thus highly desirable to explore 2D MOFs made from ligands and metal nodes that enforce non-hexagonal lattice geometries. In this article, we use first-principles calculations to computationally design metal-phthalocyanine (MPc)-based 2D MOFs with square lattices. Our study reveals rich magnetic behavior in this new class of 2D MOFs. In particular, we find that a charge-neutral MOF made from octaaminophthalocyanines metallated by Mn^2+^ ions and bound by Ni^2+^ ions through the phenylenediimine linkages should exhibit high-temperature ferromagnetic half-metallicity in monolayer form. In the bulk phase, MnPc based 2D MOFs exhibit strong intralayer but relatively weak interlayer magnetic coupling that is sensitive to the relative interlayer in-plane displacement. These results indicate the possibility of engineering the magnetic behavior of bulk or few-layer systems by controlling the interlayer stacking geometries.

## Results and discussion

### Structural and magnetic ground states of monolayers

**Fig. 2:**
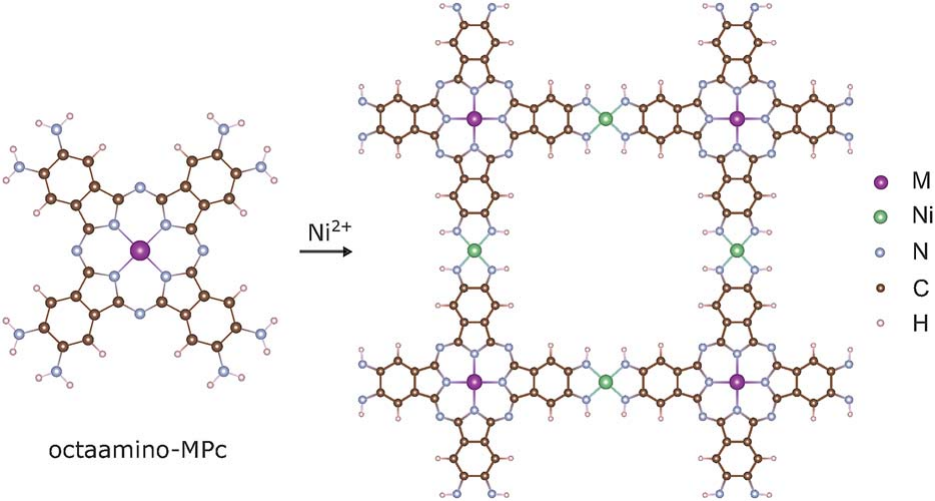
Molecular models of octaamino-metal phthalocyanine (MPc) and NiMPc two-dimensional (2D) metal–organic frameworks (MOFs). M = Cr, Mn, Fe, Co, Ni, Cu and Zn.

The MPc based 2D MOFs are constructed by conjugating 2,3,9,10,16,17,23,24-octaamino-substituted MPc molecules with Ni^2+^ ions, akin to the synthetic route of triphenylene-based 2D MOFs.^11^ In these materials, the amino groups are deprotonated and become imino groups. The nitrogen atoms of the imino groups in MPc molecules coordinate with Ni ions in square-planar geometry, resulting in long-range planar crystalline structures with square lattices, NiMPc, illustrated in Fig. 2. In this study, we considered materials containing metal-phthalocyanines (MPc) metallated with first row transition metals: M = Cr, Mn, Fe, Co, Ni, Cu, Zn. In addition to being thermally and chemically stable, these MPc molecules possess a wide range of intriguing electrical, optical and magnetic properties, which have been extensively investigated in the past.^24–26^ Despite their popularity in molecular materials, MPc moieties have not been frequently studied as components of covalent polymers.^27–29^ The rapid progress being made in synthesizing metal-ion mediated, π-conjugated 2D MOFs^8–15^ calls attention to MPc-based 2D MOFs, whose unique lattice symmetry, chemical tunability, and rich physical properties could distinguish them from other existing 2D MOFs.

Our first-principles calculations are based on density functional theory (DFT),^30,31^ as implemented in the Vienna ab initio simulation package (VASP).^32,33^ Exchange–correlation functional of the Perdew–Burke–Ernzerhof (PBE) form within the generalized gradient approximation (GGA)^34,35^ was used. Coulomb and exchange interactions of the localized d orbitals in TM elements were treated in the framework of the DFT+U method,^36^ using the Dudarev approach.^37^ The effective Coulomb (*U*) and exchange (*J*) parameters in DFT+U are *U* = 4 eV and *J* = 1 eV. This set of (*U*, *J*) parameters for TM d orbitals have been tested extensively in MPc-based systems.^28,38–40^ We have also tested a slightly different set of (*U*, *J*) parameters for MPc recently proposed by Brumboiu *et al.* ^41^ and obtained results with quantitative similarity. Other details of the computational methods can be found in the ESI.†

To determine the structural and magnetic ground states of the 2D MOFs, structural models of the 2D MOFs in unit cells were first fully relaxed in DFT+U with spin-polarized conditions. After relaxation all the systems adopt structures that can be identified as 2D square lattices in the *p*4*m* plane group. The CrPc, MnPc, FePc, CoPc and CuPc 2D MOFs exhibit unit cell magnetization of 4, 3, 2, 1, and 1 Bohr magneton (*μ*_B_) respectively. The NiPc and ZnPc-based materials are non-magnetic because the TM atoms in these MPc have closed shells.^26^ In the systems that exhibit magnetization, the magnetic moments are predominantly localized on the TM atoms in the MPc moieties. The square-planar Ni ions connecting the MPc moieties do not carry magnetic moments that can be attributed to localized d electrons, since the Ni ions do not possess unpaired electrons in the square-planar coordination mode.

**Fig. 3:**
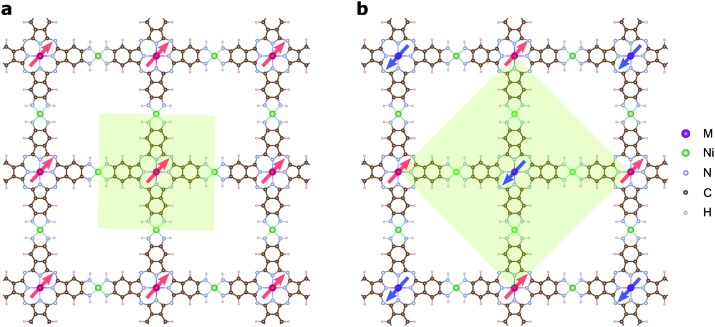
Magnetic ground states of NiMPc 2D MOFs. Red and blue arrows denote spin-up and spin-down local magnetic moments, respectively. (a) and (b) Illustrate ferromagnetic (FM) and antiferromagnetic (AFM) coupling between metal ions in MPc moieties. The shaded regions in (a) and (b) indicate the structural unit cell and the 
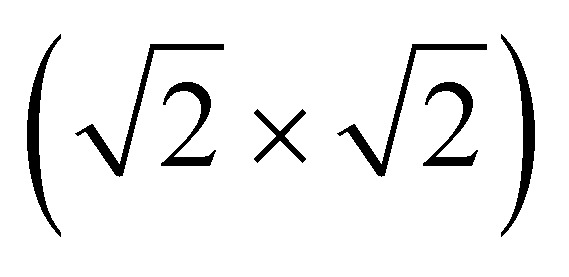
 supercell, each corresponding to the magnetic unit cell of the FM and the AFM ground states, respectively.

The magnetic ground states of the 2D MOFs with unit-cell magnetization are determined by comparing the energies of the systems with ferromagnetic (FM) or antiferromagnetic (AFM) coupling between the TM atoms in the MPc moieties. This is carried out in a 
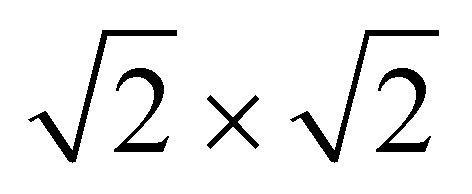
 structural supercell, which would be the magnetic unit cell if the system assumed AFM coupling, as illustrated in Fig. 3. Denoting the system energy per 
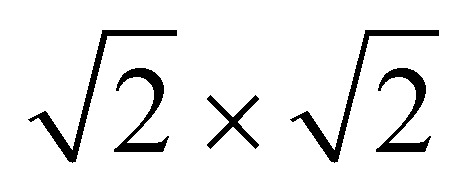
 supercell by *E*_AFM_ and *E*_FM_ for AFM and FM coupling, respectively, the magnetic exchange energy per supercell is defined as *E*_ex_ ≡ *E*_AFM_ – *E*_FM_. Accordingly, the zero-temperature magnetic ground state of the system would be FM if *E*_ex_ > 0, and AFM if *E*_ex_ < 0. In Table 1 we list the computed values of *E*_ex_ and the energy band gap corresponding to the magnetic ground states. Among the various systems investigated here, the CrPc, FePc, CoPc and CuPc-based systems are all semiconductors with weak AFM ground states. The calculated magnetic ground states of these 2D MOFs are in line with those obtained by Zhou *et al.* for MPc-based organometallic monolayers in the absence of the square-planar Ni linkages.^28^ Surprisingly, the MnPc-based system, NiMnPc, exhibits a FM metallic ground state with substantial exchange energy (*E*_ex_ = 183 meV). In contrast to the rest of the NiMPc materials considered here, the exchange energy of NiMnPc, 183 meV, is significantly larger than the MnPc monolayer system studied by Zhou *et al.*, 62 meV (40 meV using our calculation setups), when evaluated in the 
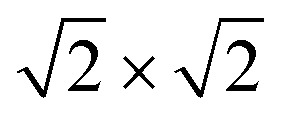
 superlattice. In the following, we investigate the origin of the unusually strong FM coupling in the NiMnPc monolayer system.

**Table 1:** Total magnetic moments per unit cell (*M*), exchange energies per 
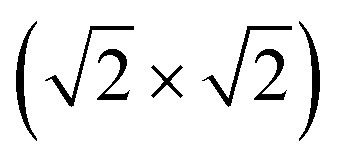
 supercell (*E*_ex_), and energy band gaps (*E*_g_) of different MPc based 2D MOFs calculated using DFT+U

	Cr	Mn	Fe	Co	Ni	Cu	Zn
*M* (*μ*_B_)	4	3	2	1	0	1	0
*E*_ex_ (meV)	–36	183	–20	–19	—	–0.4	—
*E*_g_ (eV)	0.35	Half-metal	0.28	0.35	0.35	0.30	0.30

**Fig. 4:**
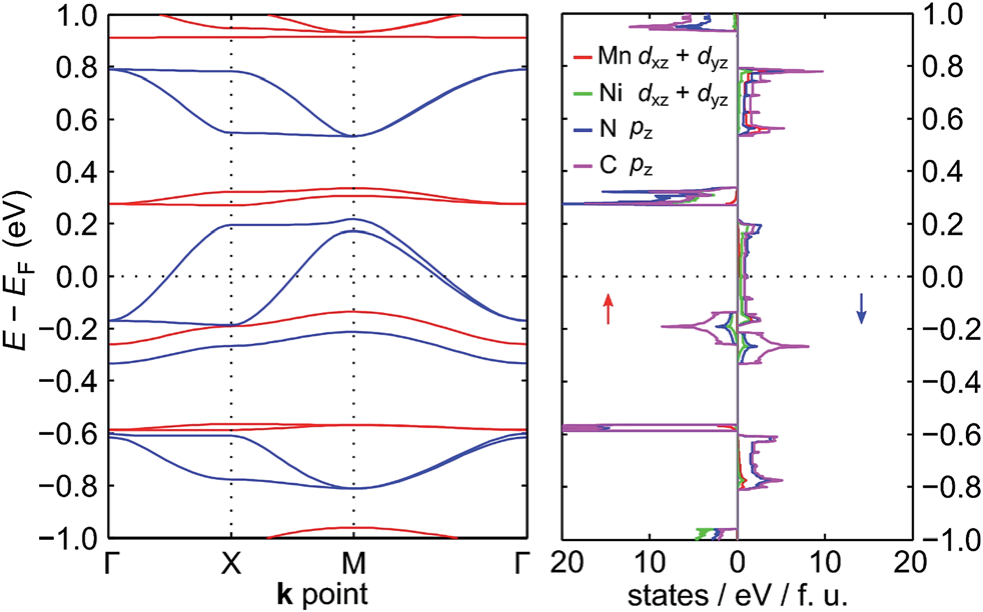
Electronic band structure and partial density of states (PDOS) of NiMnPc near the Fermi level. In the left panel, the spin-up bands are in red, and the spin-down bands are in blue. The states near the Fermi level are mainly derived from π orbitals, which include the d_*xz*_ + d_*yz*_ orbitals of Mn and Ni, and the p_*z*_ orbitals of N and C. The PDOS of these orbitals are shown in the right panel.

The electronic band structure of NiMnPc near the Fermi level and the corresponding partial density of states (PDOS) are shown in Fig. 4 (the band structures of the other NiMPc 2D MOFs are shown in Fig. S1†). Notably, the electronic states of NiMnPc in the vicinity of Fermi level are completely spin-polarized. The system is therefore a half-metal whose low-energy electronic phenomena would be dominated by one type of spin-polarized electrons, which could be relevant for spintronic applications. Analysis of the PDOS of bands near the Fermi level reveals that nearly all the bands are derived from the hybridization of π orbitals, namely the symmetry-equivalent d_*xz*_ and d_*yz*_ orbitals of Mn and Ni, as well as the p_*z*_ orbitals of N and C. In combination with the metallic nature of the system, it can be concluded that the π orbitals of the system are delocalized. We note that because the electronic bands near the Fermi level are relatively dispersive, the MnPc based 2D MOFs should also have good electrical conductivity, a highly sought-after property for this class of materials.^17^

### Mechanism of magnetic exchange

**Fig. 5:**
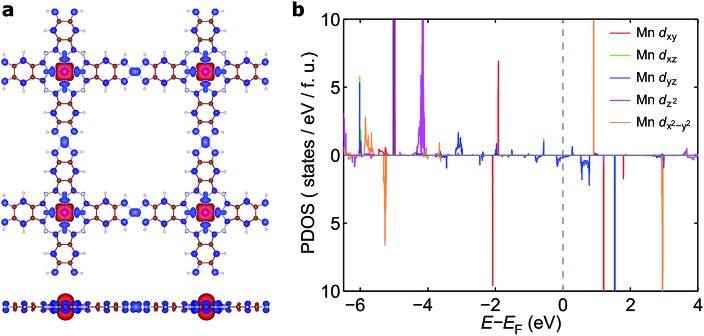
Spin density isosurface of NiMnPc and PDOS of Mn d orbitals. (a) Top and side view of the spin density isosurface at absolute spin-density value |*ρ*_↑_ – *ρ*_↓_| = 0.01 electrons per Å^3^. Red and blue isosurfaces correspond to positive and negative spin density, respectively. (b) PDOS of Mn d_*xy*_, d_*xz*_, d_*yz*_, d_*z*^2^_, d_*x*^2^–*y*^2^_ orbitals. The PDOS of symmetry-equivalent d_*xz*_ and d_*yz*_ overlap with each other.

**Fig. 6:**
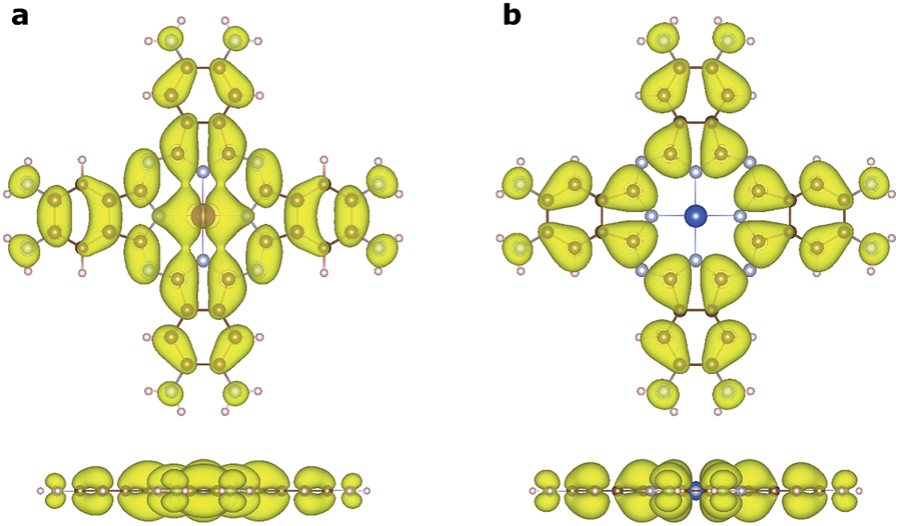
Visualization of the highest occupied molecular orbitals (HOMOs) of octaamino-MPc molecules by charge density isosurfaces. (a) Strong hybridization is evident between the d_π_ orbitals of Mn and the π orbitals of the ligand in octaamino-MnPc. (b) No contribution is observed from the metal d orbitals in the HOMO charge density isosurface of octaamino-MPc, where M is Cr, Fe, Co, Ni, Cu, or Zn. Both top and side views of the charge density isosurfaces (*ρ* = 0.001 electrons per Å^3^) are shown.

To understand the nature of the unusually strong FM coupling in NiMnPc, we plotted the spin density isosurface of the system and the PDOS of Mn d orbitals in Fig. 5. As shown in Fig. 5a, positive spin density (*ρ*_↑_ – *ρ*_↓_ > 0) mainly appears on the Mn atoms, and is associated with the localized d electrons. The adjacent nitrogen atoms that bond directly with Mn are oppositely polarized. Notably, the electron densities surrounding the square-planar Ni–N moieties and carbon atoms are also slightly polarized with an oscillatory character in real space. The spin polarization of these bridging units can be attributed to the polarization of the delocalized π orbitals as shown in Fig. 4. The PDOS of the Mn d orbitals in Fig. 5b shows that, except for a portion of the d_*xz*_/d_*yz*_ orbitals that hybridize with the π orbitals of other atoms, the d orbitals of the Mn generally have very small dispersion, consistent with their localized nature. Given the large distance between the Mn atoms in neighboring MnPc moieties (∼18.3 Å), direct exchange between the d orbitals of Mn is rather weak, and thus cannot be responsible for the strong FM coupling between the Mn atoms. We conclude instead that the coupling occurs through the indirect exchange *via* the metallic π orbitals. Namely, the localized spin moments of the Mn atoms in individual MnPc moieties polarize the delocalized π orbital through direct exchange interaction. The π orbitals further mediate FM coupling of the Mn atoms at neighboring sites, as FM coupling between different MnPc moieties results in π orbitals with a consistent sign of polarization. The latter facilitates the hybridization of the π orbitals on different MnPc moieties and reduces the energy of the system. This indirect exchange through delocalized electrons is similar to the Zener d–p exchange^42^ or the RKKY^43–45^ exchange mechanism. One possible reason why this mechanism is operative in NiMnPc is that the d_*xz*_/d_*yz*_ orbitals of Mn participate in the hybridization of π orbitals, leading to metallicity, stronger exchange interaction between the localized d orbitals and the π orbitals, and hence FM coupling. In contrast, in other NiMPc 2D MOFs, including CrPc, FePc, CoPc, NiPc, CuPc and ZnPc based materials, the filling of the d orbitals of TM atoms, and their energy alignment with respect to the π orbitals of the phthalocyanine ring and the linking Ni atoms, are not conducive to orbital hybridization. The localized magnetic moments of the TM atoms can therefore only interact through weak super-exchange or direct exchange, leading to AFM ground states with small exchange energies. The underlying cause of this difference in d electron hybridization can be clearly seen in the computed highest occupied molecular orbital (HOMO) of octaamino-MPc molecules, as shown in Fig. 6. Importantly, among the octaamino-MPc molecules, only the HOMO of MnPc has substantial contribution from the d orbitals of the TM atom and is fully delocalized over the entire molecule. This unique feature of strong hybridization between the metal d orbitals and the π electrons of ligand in octaamino-MnPc also occurs in amino-free MnPc molecules, as has been demonstrated both experimentally and theoretically.^41,46–48^

The square-planar Ni–N moieties in the MnPc-based 2D MOFs also play a key role in the strong FM exchange between the Mn atoms by effectively mediating the π electron conjugation in different octaamino-MPc molecules. This is consistent with the fact that 2D MOFs with some of the highest measured electrical conductivity were realized by π-conjugation through square-planar Ni–N linkage.^9,11^ We also find that replacing the nitrogen linker atoms in the square-planar Ni–N moieties by sulfur or oxygen does not significantly increase or decrease the magnetic exchange energy, suggesting that catechol and thiocatechol moieties may also mediate effective π electron conjugation (see also Fig. S2†).

### Monte-Carlo simulation of ferromagnetic phase transition

At elevated temperature, entropy favors disordered alignment of magnetic moments, causing NiMnPc to undergo a FM to paramagnetic phase transition at the Curie temperature, *T*_c_. A critical issue affecting the theoretical determination of *T*_c_ is constructing an appropriate Hamiltonian that captures the nature of magnetic interactions in the system. The simplest model that describes isotropic interactions between local magnetic moments is the Heisenberg model 
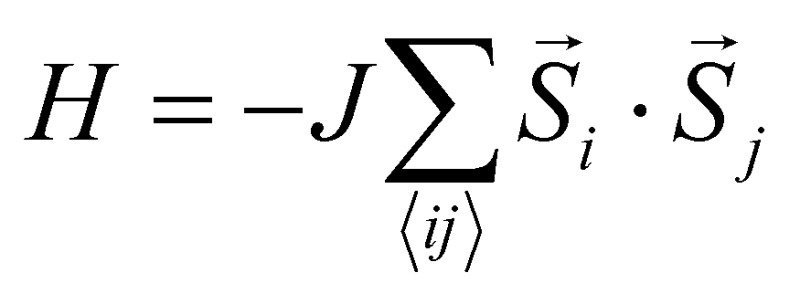
, where *S*_*i*_ and *S*_*j*_ are the spin operators of magnetic moments at neighboring site *i* and *j*, and *J* is the exchange constant. However, it is well-known that in two dimensions, the isotropic Heisenberg model does not have spontaneous symmetry breaking at non-zero temperature due to the divergent contributions of gapless spin-waves.^49^ In reality, magnetic anisotropy due to spin–orbit coupling and/or magnetic dipolar interactions almost always exists, such that long-range magnetic order is usually observed with the addition of even a small amount of anisotropy.^50–55^ An extreme case is the 2D Ising model 
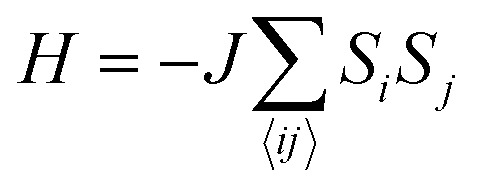
, which corresponds to infinitely large magnetic anisotropy, such that the spin vectors can only point in the *z*-direction (*S*_*i*_ = +*S* or –*S*). Accordingly, the Ising model leads to FM or AFM ground states at non-zero temperature. Even though it has been used widely to calculate the *T*_c_ of 2D magnetic systems,^28,56,57^ it is not yet clear to what extent the Ising model overestimates *T*_c_ in most experimental situations, where there is a finite amount of magnetic anisotropy.

We attempt to predict more realistic *T*_c_ values for 2D FM systems by using two models that consider finite magnetic anisotropy. The first one (hereafter referred as Model 1) is the anisotropic Heisenberg model, which incorporates magnetic anisotropy by exchange anisotropy:1
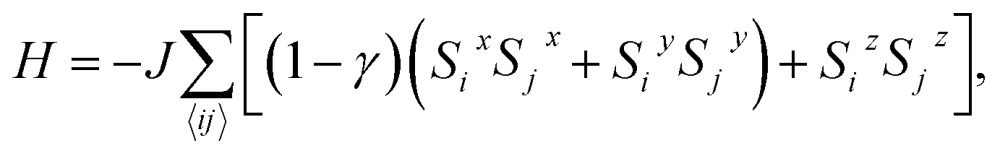
where *S*_*i*_ = (*S*_*i*_^*x*^, *S*_*i*_^*y*^, *S*_*i*_^*z*^) denotes a unit vector of a classical magnetic moment at site *i*, the sum *ij* is over nearest neighbor pairs, *J* is the magnetic exchange constant, and *γ* is a dimensionless parameter that characterizes the strength of exchange anisotropy. For FM coupling between the spins, the values of *J* and (1 – *γ*) are positive. The magnetic “easy axis”, along which the exchange interaction is stronger, is chosen to be the *z* direction. The exchange anisotropy parameter *γ* can therefore take any values between 0 and 1. For *γ* = 0, the 2D isotropic Heisenberg model is obtained; *γ* = 1 gives an Ising-like model, that differs from the classical 2D Ising model in that the magnetic moments are still allowed to point in any arbitrary direction, not just + *S* or –*S*.

In Model 2, we assume isotropic exchange interactions, but take into consideration the presence of uniaxial single-ion anisotropy. This model enforces an energy penalty on magnetic moments pointing to directions other than the magnetic easy axis (chosen to be the *z* axis), irrespective of the spins at neighboring sites:2
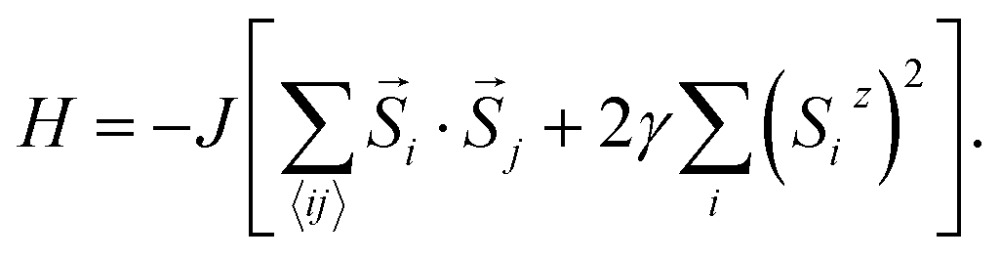



In this model, the strength of magnetic anisotropy, represented by *γ*, can take any positive values. A prefactor 2 is added before *γ* to facilitate comparison between Model 1 and Model 2, based on the energy cost of rotating the spins. The case *γ* = 0 in this model again corresponds to the 2D isotropic Heisenberg model, whereas the case *γ* = +∞ represents the classical 2D Ising model, since spin components in directions other than the magnetic easy axis are now fully penalized.

**Fig. 7:**
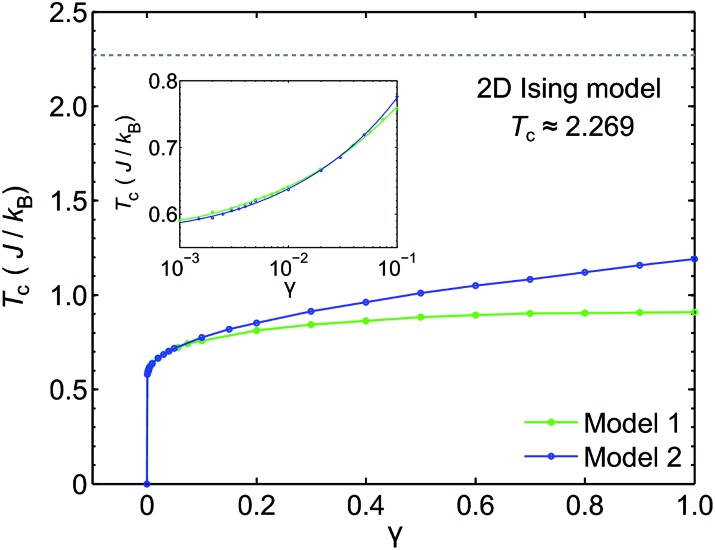
The Curie temperature (*T*_c_) of 2D spin models in square lattices as a function of magnetic anisotropy parameter *γ*. The results for Model 1 (2D Heisenberg model with exchange anisotropy) and Model 2 (2D Heisenberg model with single-ion anisotropy) are plotted together. The inset shows *T*_c_ at small values of *γ* using log-scale horizontal axis. The *T*_c_ projected using the classical 2D Ising model is shown as a dashed line.

We use classical Monte Carlo simulations to determine *T*_c_ with Model 1 and Model 2 as a function of the magnetic anisotropy parameter *γ*. *T*_c_ is expressed as a function of *J*/*k*_B_, where *k*_B_ is the Boltzmann constant. The Monte Carlo simulations were performed using the Metropolis algorithm, and *T*_c_ was determined by finding the intersection of Binder cumulants^58^ for two system sizes (40 × 40 and 80 × 80, consisting of 1600 and 6400 spins respectively). The calculated *T*_c_ as a function of *γ* for Model 1 and Model 2 are plotted in Fig. 7. Our simulations confirm previous studies^52–55^ that even a small amount of magnetic anisotropy can stabilize a FM ground state at finite temperature in the 2D Heisenberg model. When the values of *γ* are small (*i.e.* 0.001 < *γ* < 0.1), the *T*_c_ values determined by Model 1 and Model 2 were found to be close, as shown in the inset of Fig. 7. However, when *γ* > 0.1, the *T*_c_ predicted by Model 2 becomes noticeably larger. Because the value of *γ* in Model 1 is restricted in the range between 0 and 1, the maximum *T*_c_ of this model is reached at *γ* = 1.0, which gives *T*_c_ ≈ 0.9*J*/*k*_B_. In comparison, Model 2 predicts *T*_c_ ≈ 1.2*J*/*k*_B_ at *γ* = 1.0. Importantly, in the range of *γ* from 0 to 1, the *T*_c_ predicted by both models are significantly lower than that of the 2D Ising model, which has an analytical solution, 
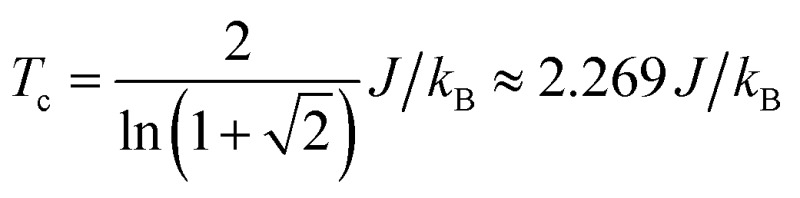
.^59^ For Model 2, the Ising limit is only asymptotically reached at large values of *γ* (*γ* > 100, see Fig. S3†). Hence, caution must be exercised when using the classical Ising model to predict the *T*_c_ of 2D magnetic systems. Indeed, our results here show that in the absence of particularly strong magnetic anisotropy, the Ising model significantly overestimates *T*_c_. Although quite dramatic, this overestimation is not surprising because in the Ising model the size of the accessible phase space for thermal excitation is significantly smaller compared to continuous spin models, low-energy excitations such as spin-waves being absent in the Ising model.

With a model that more comprehensively considers magnetic anisotropy in hand for magnetic phase transitions in the 2D space, we calculated the *T*_c_ of NiMnPc on the basis of the model parameters obtained from first-principles calculations. The exchange constant *J* in this system is related to the exchange energy *E*_ex_ evaluated in the 
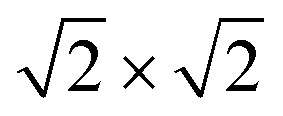
 supercell by *J* = (1/8)*E*_ex_, which gives *J* = 22.9 meV. The magnetic anisotropy constant *γ* can be evaluated by relating it to the exchange constant *J* and the magnetic anisotropy energy (MAE), the latter of which can calculated from DFT+U with spin–orbit coupling.^60^ The value of MAE is determined as MAE ≡ *E*_∥_ – *E*_⊥_, where *E*_∥_ and *E*_⊥_ represent the energies of the system when the spin moment is parallel or perpendicular to the 2D plane of the MOF monolayer, respectively. A positive MAE implies that out-of-plane spin alignment is energetically more favorable than in-plane alignment. Our calculation gives MAE = 0.74 meV for a unit cell of NiMnPc monolayer. Although it is possible to specifically calculate the contribution of both exchange anisotropy and single-ion anisotropy to MAE from first principles, we have shown in Fig. 7 that the *T*_c_ values predicted by Models 1 and 2, corresponding to exchange anisotropy and single-ion anisotropy, respectively, are similar for small *γ*. In both models, the calculated MAE is related to *γ* through MAE = 2*γJ*. This gives *γ* = 0.017, which corresponds to *T*_c_ ≈ 0.66*J*/*k*_B_, or 170 K. In contrast, the frequently used 2D Ising model, where *T*_c_ ≈ 2.269*J*/*k*_B_, gives a transition temperature value higher than 600 K. Notably, when the square-planar NiN_4_ moiety in NiMnPc is changed to NiO_4_ or NiS_4_ (*i.e.*, the phenylenediamine units are changed to catechols or thiocatechols) the predicted *T*_c_ are approximately 230 K and 150 K, respectively. Although the *T*_c_ values for the NiN_4_, NiO_4_, and NiS_4_ analogues of MnPc-based MOFs predicted using our realistic anisotropy model are still below room temperature, they sit above the liquid nitrogen temperature and thus present significant potential for practical applications (we note that we have ignored the phonon excitation and quantum fluctuation effects in calculating the *T*_c_). Growth of these 2D MOFs as single layers on appropriate substrates, as might be expected under experimental conditions, could act as additional symmetry-breaking measures to enhance the magnetic anisotropy,^61–63^ further increasing *T*_c_.

### Bulk structural and magnetic properties

**Fig. 8:**
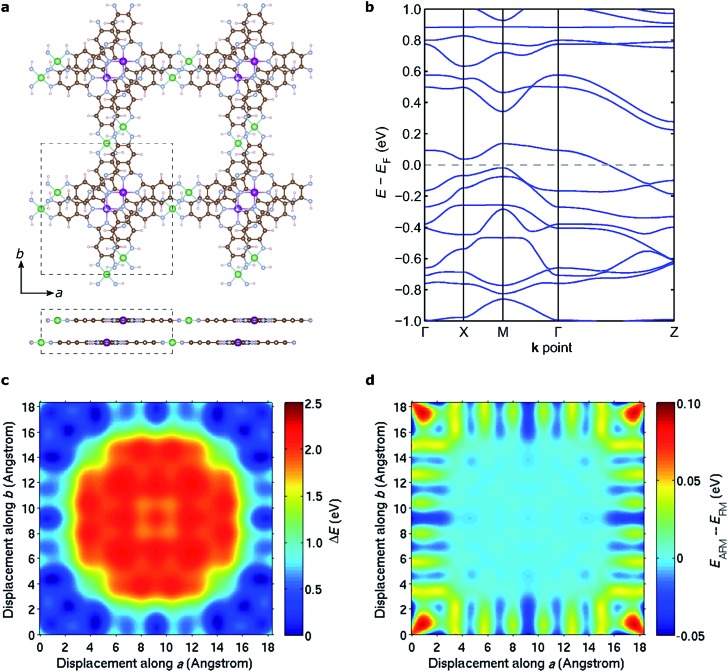
Stacking mode and interlayer magnetic coupling in bulk NiMnPc. (a) Molecular structure of the lowest-energy stacking configuration as determined by DFT calculations. The lowest-energy configuration has interlayer distance ∼3.1 Å, and exhibits relative in-plane displacements of ∼2.3 Å along both ***a*** and ***b*** axes between adjacent layers. The dashed boxes in the top and side views outline the tetragonal unit cell of the bulk structure. (b) Calculated electronic band structure of the lowest-energy stacking configuration. (c) Potential energy surface generated by relative displacement of the two layers in the unit cell along ***a*** and ***b*** axes, while fixing the interlayer distance at the value of lowest-energy configuration. (d) Variation of the energy difference between interlayer AFM and FM coupling (*E*_AFM_ – *E*_FM_) while the two layers are displaced as in (c).

The discussion above pertains to single layers of 2D MOFs. The experimental realization of such single-layer structures notwithstanding, 2D MOFs synthesized thus far form bulk structures, where multiple monolayers stack to form layered bulk structures. To determine the most favorable interlayer stacking for NiMnPc, we considered two potential symmetries for bulk lattices: monoclinic and tetragonal (see Fig. S4†). For the monoclinic lattice, the bulk unit cell contains one monolayer, with perpendicular ***a***, ***b*** lattice vectors residing in the monolayer plane, and the out-of-plane ***c*** vector determining the interlayer separation and the relative (***ab***) displacement between neighboring layers. In the tetragonal lattice, we considered unit cells containing two monolayers with different relative (***ab***) displacements. In this case, the ***c*** vector is always normal to the (***ab***) plane and thus determines only the interlayer separation, not the relative layer displacement. Each of these unit cells gives rise to a large number of possible bulk stacked structures with different interlayer separations and relative in-plane displacements. We started from the relaxed structure of each monolayer and used DFT+U in combination with van der Waals correction in the form of DFT-D2 (ref. 64) to calculate the system energy for bulk models. In doing so, we considered both FM and AFM interlayer coupling for all structures and found that the lowest energy configuration adopts a tetragonal lattice with interlayer separation of ∼3.1 Å and relative in-plane displacement of ∼2.3 Å along both the ***a*** and ***b*** axes, as illustrated in Fig. 8a. The interlayer magnetic coupling was found to be weakly AFM, with exchange energy *E*_ex_ ≈ –6 meV per unit cell. Notably, band structure calculations suggest that this lowest-energy bulk lattice is expected to be metallic (Fig. 8b); the experimental realization of this material would thus contribute a rare example of a highly electrically conductive MOF.^17^ Fig. 8c shows the potential energy surface of NiMnPc as a function of the relative in-plane displacement between two neighboring layers in the tetragonal unit cell, with a fixed interlayer separation of 3.1 Å. The complex energy landscape results from a competition between the energy cost of orbital overlap between orbitals on neighboring layers (especially the Mn orbitals) and the energy gain from enhanced van der Waals interactions when neighboring layers have small relative in-plane displacement, as can be seen from Fig. S5.† Expectedly, the interlayer magnetic coupling is itself sensitive to the relative in-plane displacement because the orbitals mediating magnetic coupling also vary with the displacement. This gives rise to oscillatory FM or AFM ground states depending on the relative displacement (Fig. 8d). Taken together, our results suggest that bulk NiMnPc should exhibit strong intralayer and relatively weak interlayer magnetic coupling that are sensitive to the stacking mode, portending the possibility that the magnetic behavior of bulk or few-layer systems can be rationally controlled by varying the interlayer stacking geometry.

## Conclusions

We demonstrate using first-principles calculations that metal phthalocyanine-based 2D MOFs conjugated by square-planar nickel ions exhibit rich structural and magnetic behavior. In particular, the MnPc-based materials are expected to exhibit half-metallicity and ferromagnetic ordering below 170 K, a remarkably large Curie temperature for a monolayer material. We demonstrate that such strong magnetic coupling results from the strong hybridization between the π-symmetry orbitals of Mn and the phthalocyanine ring, with the square-planar NiN_4_ moieties playing a key role in facilitating efficient magnetic exchange and π electron delocalization. We have also devised new theoretical means to include magnetic anisotropy in evaluating *T*_c_ in 2D magnetic systems. In particular, we show that in the absence of strong magnetic anisotropy, the widely used 2D Ising model can lead to overestimation of *T*_c_ by several times. These results suggest that π electron conjugation between magnetic ions and ligand groups, together with enhanced magnetic anisotropy through the incorporation of heavy elements with sizable spin–orbit coupling, are effective strategies to design ferromagnetic 2D MOFs with high *T*_c_. The materials studied in this work are based on existing molecular building blocks and we are thus confident that they could be realized experimentally. Indeed, synthetic methods for octaaminophthalocyanine exist,^65^ and the square-planar conjugation of phenylenediamine linkers with Ni is also well documented and has already been used to make highly conductive 2D MOFs.^11^ Considering the tremendous amount of chemical tunability in the MPc-based 2D MOFs stemming from metal replacement^24,25^ and the utilization of different linking groups and ligating *D*_4h_ metal ions,^17^ the new class of square lattices proposed here represents a rich platform for new solid-state physics phenomena in MOFs, with the promise of a wide range of potential applications in spintronics and electronics.
