# Supplementary material for: High temperature ferromagnetism in π-conjugated two-dimensional metal–organic frameworks
Source: Chem Sci. 2017 Feb 8;8(4):2859–67. doi: 10.1039/c6sc05080h (PMC5428024; doi:10.1039/c6sc05080h)
Supplement: Supplementary file 1 [file SC-008-C6SC05080H-s001.pdf]

Electronic Supplementary Material (ESI) for Chemical Science.
This journal is © The Royal Society of Chemistry 2017

Electronic Supplementary Information for:

High Temperature Ferromagnetism in π-Conjugated Two-Dimensional Metal-Organic Frameworks

Wenbin Li[†], Lei Sun[‡], Jingshan Qi[§], Pablo Jarillo-Herrero[‖], Mircea Dincă[*,‡], and Ju Li[*,§]

[†]Research Laboratory of Electronics, Massachusetts Institute of Technology, Cambridge, Massachusetts 02139, United States

[‡]Department of Chemistry, Massachusetts Institute of Technology, Cambridge, MA 02139, United States

[§]Department of Nuclear Science and Engineering and Department of Materials Science and Engineering, Massachusetts Institute of Technology, Cambridge, MA 02139, United States

[‖]Department of Physics, Massachusetts Institute of Technology, Cambridge, Massachusetts 02139, United States

*Corresponding Authors. Email: mdinca@mit.edu (M.D.); liju@mit.edu (J.L.)

Table of Contents

Computational Methods **S2**

Figure S1 | Electronic band structures of different NiMPc 2D MOFs **S3**

Figure S2 | NiMnPc 2D MOFs with square-planar NiO_4 or NiS_4 moieties **S4**

Figure S3 | T_c vs. γ for 2D Heisenberg model with single-ion anisotropy **S5**

Figure S4 | Structural models of bulk NiMnPc **S6**

Figure S5 | Contributions to bulk energy change during interlayer displacement **S7**

Supplementary References **S8**

Computational Methods

Density functional theory (DFT) calculations were carried out using the Vienna ab initio Simulation Package (VASP)[1,2], version 5.3.3. The electronic wavefunctions were expanded in a plane-wave basis set with a kinetic energy cutoff of 500 eV. Electron-ion interactions were described using the projector augmented wave (PAW) method[3,4]. Generalized gradient approximation (GGA)[5] of the exchange-correlation energy in the form of Perdew-Burke-Ernzerhoff (PBE)[6] was applied. We used the Dudarev implementation of DFT+U method[7,8] to describe the localized *d* orbitals of transition metal atoms. The effective Coulomb (*U*) and exchange (*J*) parameters being used are described in the main text. All calculations were carried out in spin-polarized conditions[9]. The initial magnetic moments of the transition metal atoms were set to be their corresponding electronic magnetic moments in the non-bonding atomic limit, using the MAGMOM tag in VASP. We find that the relaxed magnetic moments and energies for most of the systems studied in this work does not depend on the initial magnetic moments being supplied. One exception is the NiFePc MOF system, for which convergence was found to be exceptionally difficult. In this case, we started the spin-polarized calculations with the charge density obtained from converged non-spin-polarized calculations.

The MOF monolayers were modelled by adding sufficiently large vacuum space along the out-of-plane direction in the structural model (20 Å for 1×1 structural unit cell and 15 Å for $\sqrt{2} \times \sqrt{2}$ supercell). We adopted the Monkhorst-Pack[10] scheme of **k**-point sampling for Brillouin zone integration, with $6 \times 6 \times 1$ and $4 \times 4 \times 1$ Γ-centered grid for unit cell and $\sqrt{2} \times \sqrt{2}$ supercell respectively. These **k**-points were sufficient to achieve a convergence in total energy less than 0.05 meV per atom. For bulk systems, a 6×6×5 **k**-point grid was employed. Gaussian smearing of 50 meV was used during structural optimization, whereas for total energy calculations we employed the more accurate tetrahedron method with Blöchl correction[11]. During structural relaxation all symmetry constraints were turned off. The energy convergence thresholds for the electronic and ionic relaxation degrees of freedom were 10^{-6} and 10^{-5} eV respectively. The relaxed atomic configurations of different NiMPc MOF monolayers in the POSCAR format of VASP are included separately as Supplementary Data.

Supplementary Figures

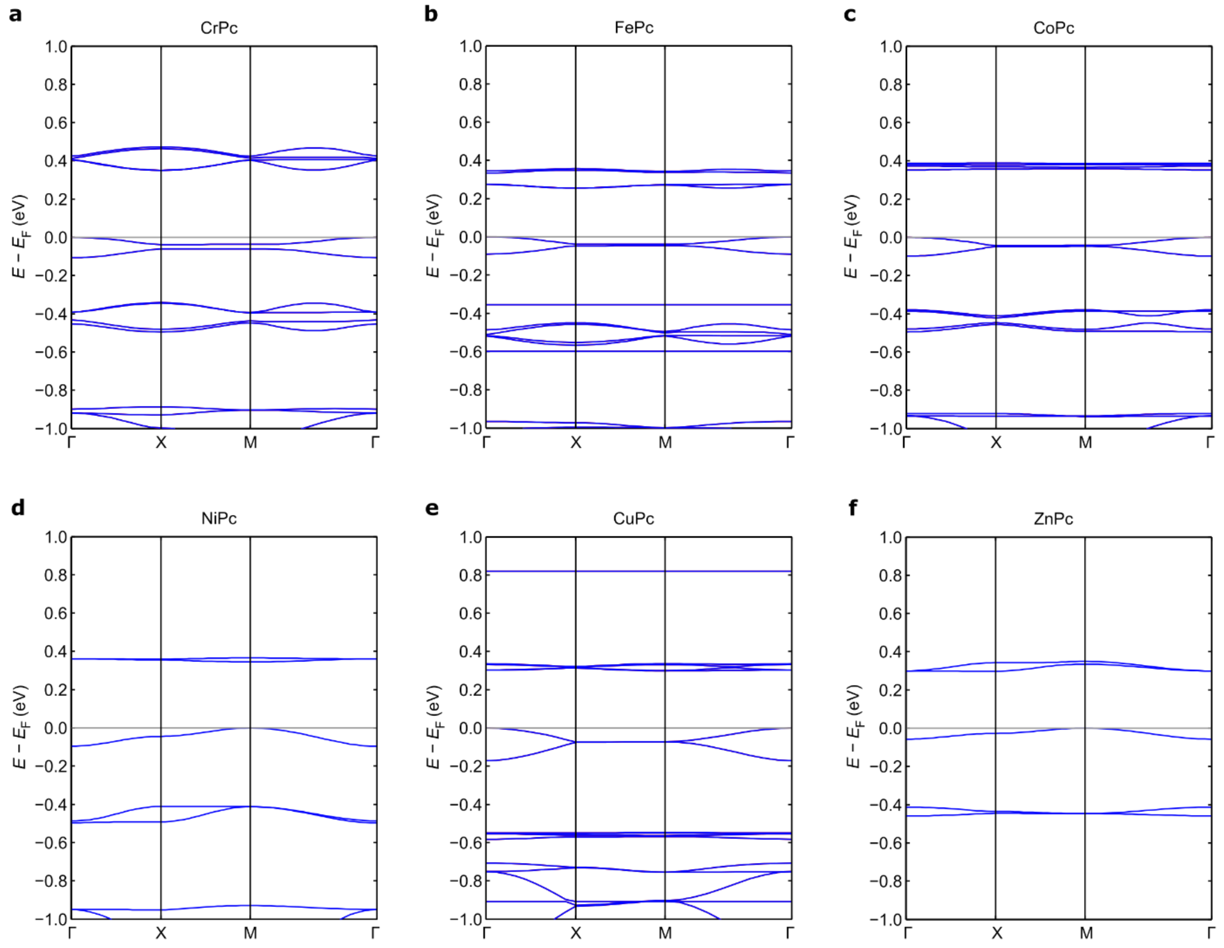

Figure S1 | Electronic band structures of different NiMPc 2D MOFs with square-planar NiN_4 moieties. (a-f) show the band structures for CrPc, FePc, CoPc, NiPc, CuPc and ZnPc based NiMPc MOF monolayer systems respectively.

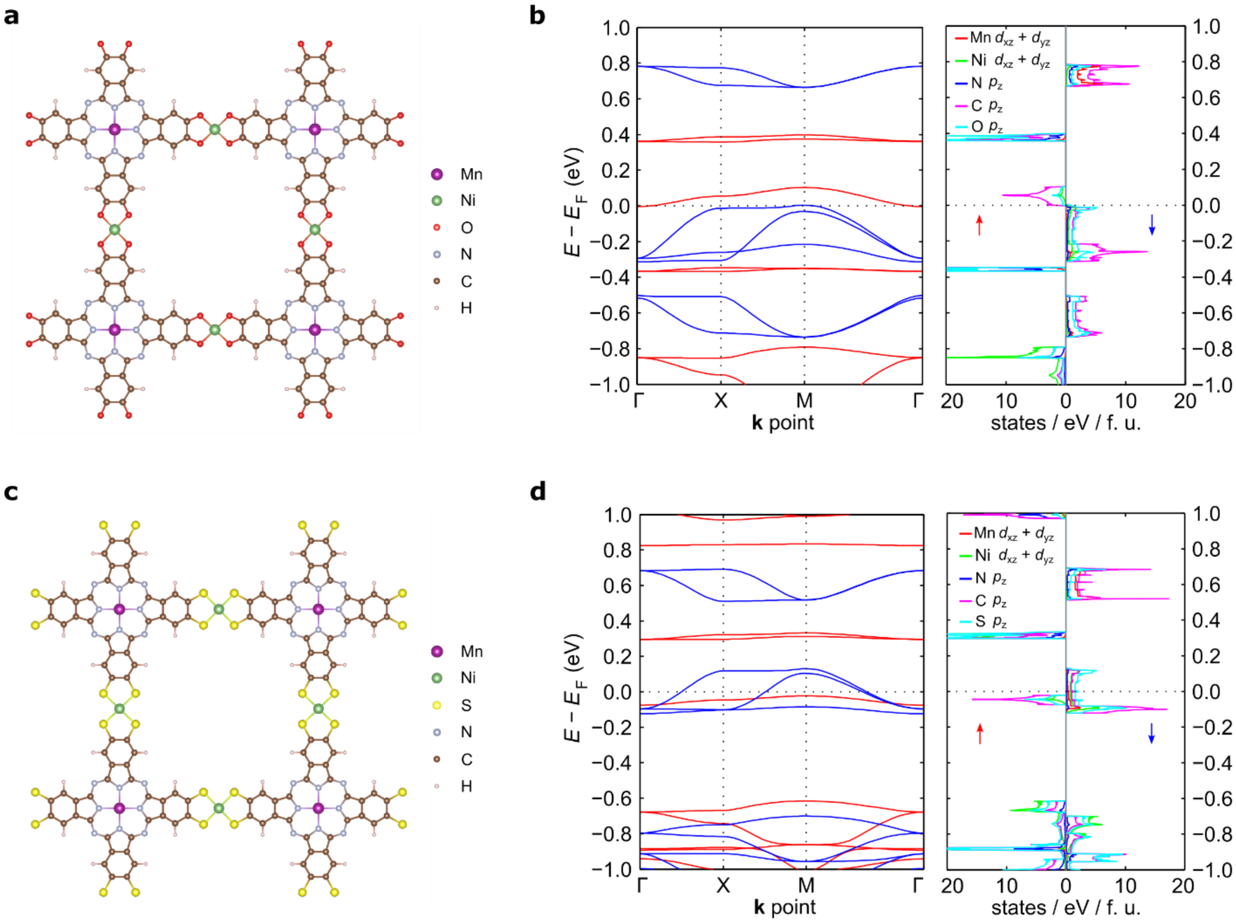

Figure S2 | DFT-relaxed atomistic configuration, electronic band structure and the corresponding PDOS of NiMnPc 2D MOFs with square-planar NiO_4 or NiS_4 moieties. In (**a-b**), the linker atoms in the square-planar moieties are oxygen, and in (**c-d**), the linker atoms are sulfur. Both systems have ferromagnetic ground states. The exchange energy evaluated in the $\sqrt{2} \times \sqrt{2}$ structural supercell is 279 meV and 168 meV for the system with oxygen and sulfur linkers respectively. The magnetic anisotropy energy was each determined to be 0.57 meV and 0.73 meV in unit cell.

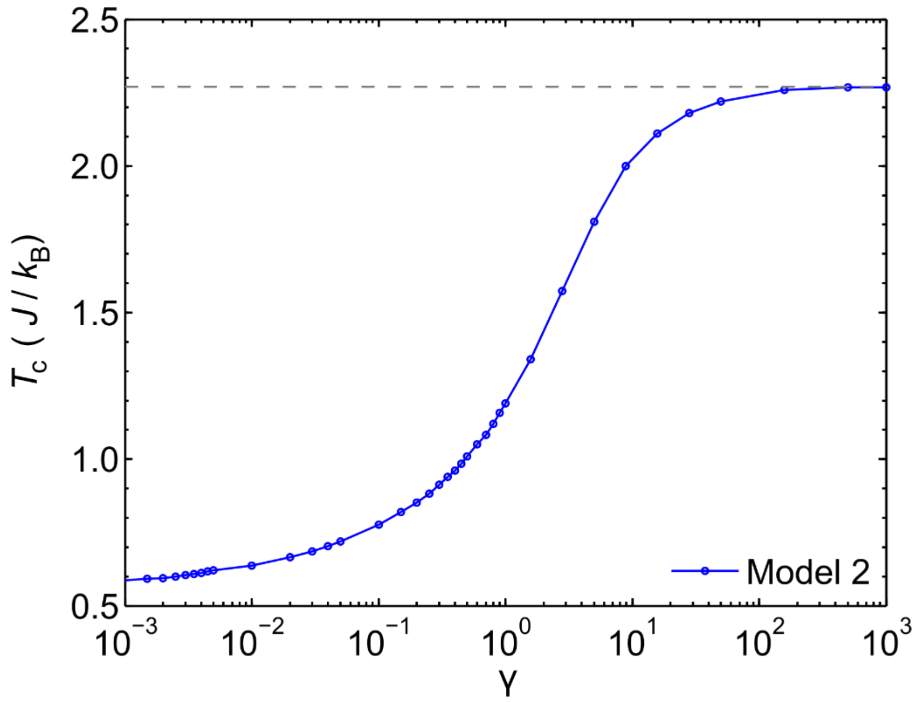

Figure S3 | Ferromagnetic transition temperature T_c as a function of magnetic anisotropy parameter γ for 2D Heisenberg model with single-ion anisotropy. The dashed line denotes the T_c of 2D Ising model ($T_c \approx 2.269\ J/k_B$).

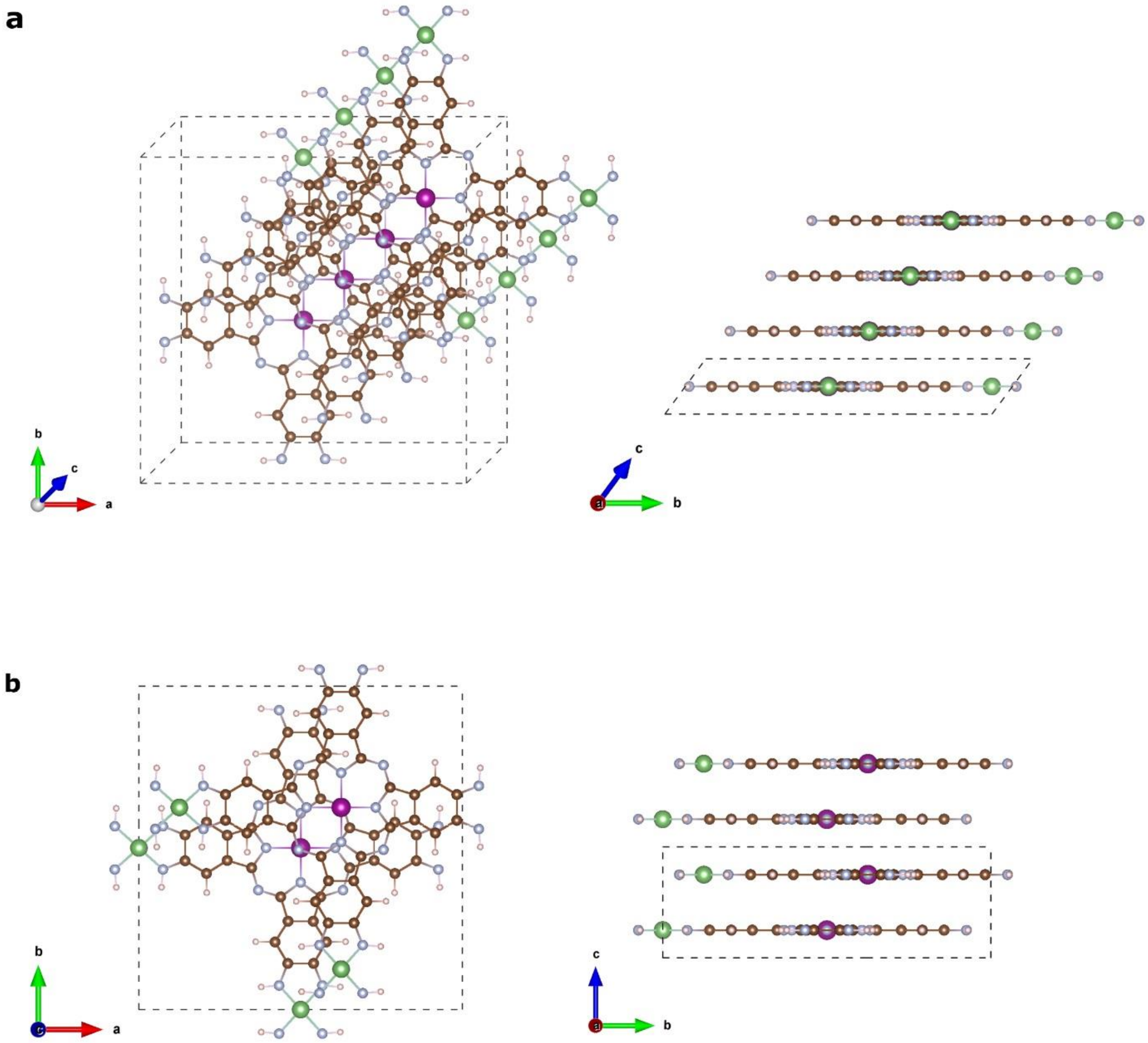

Figure S4 | Structural models of bulk NiMnPc. **(a)** Top and side views of bulk stacking configuration in monoclinic lattice. Each unit cell, as outlined in the dashed lines, includes one layer of NiMnPc. **(b)** Bulk stacking mode corresponding to tetragonal lattice. Each unit cell has two layers with relative in-plane displacement.

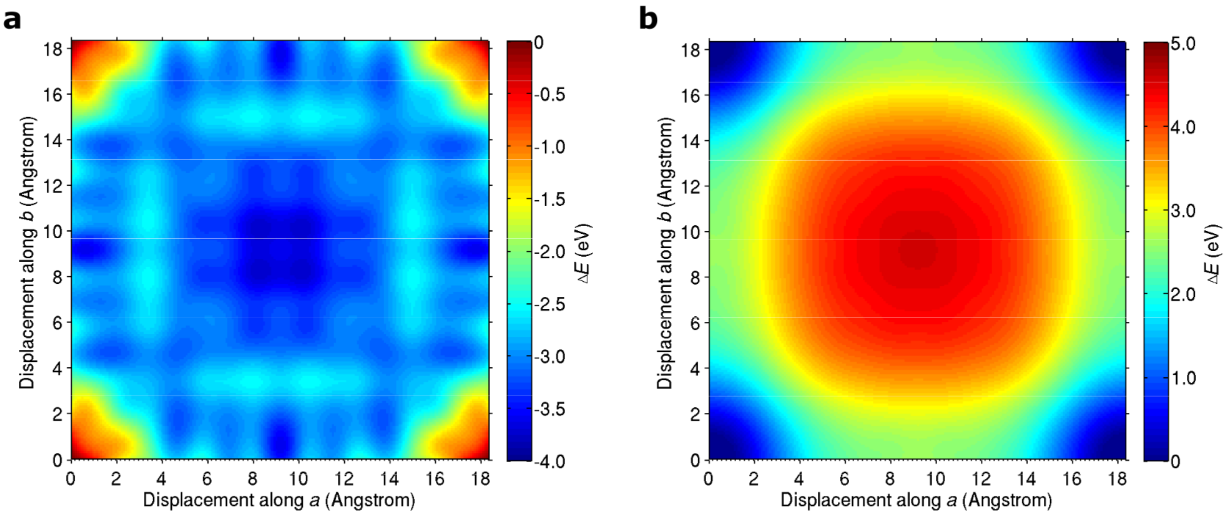

Figure S5 | Contributions to bulk system energy change from (a) interlayer hybridization effect and (b) van der Waals interaction, as two NiMnPc MOF monolayers are shifted with respect to each other along the ***a*** and ***b*** axes. The tetragonal unit cell of the bulk is the same as the one shown in the **Figure 8a** of main text, and interlayer separation is fixed at 3.1 Å. The reference energy in both (**a**) and (**b**) is the energy at zero displacement, namely, when the two layers have fully eclipsed AA stacking sequence. To facilitate interpretation of data, the interlayer magnetic coupling was fixed to be antiferromagnetic (AFM) in this case.

Supplementary References

1 Kresse, G. & Furthmüller, J. Efficiency of ab-initio total energy calculations for metals and semiconductors using a plane-wave basis set. *Comput. Mater. Sci.* **6**, 15-50 (1996).

2 Kresse, G. & Furthmüller, J. Efficient iterative schemes for ab initio total-energy calculations using a plane-wave basis set. *Phys. Rev. B* **54**, 11169-11186 (1996).

3 Blochl, P. E. Projector augmented-wave method. *Phys. Rev. B* **50**, 17953-17979 (1994).

4 Kresse, G. & Joubert, D. From ultrasoft pseudopotentials to the projector augmented-wave method. *Phys Rev B* **59**, 1758-1775 (1999).

5 Perdew, J. P. *et al.* Atoms, molecules, solids, and surfaces: Applications of the generalized gradient approximation for exchange and correlation. *Phys. Rev. B* **46**, 6671-6687 (1992).

6 Perdew, J. P., Burke, K. & Ernzerhof, M. Generalized gradient approximation made simple. *Phys. Rev. Lett.* **77**, 3865-3868 (1996).

7 Anisimov, V. I., Aryasetiawan, F. & Lichtenstein, A. I. First-principles calculations of the electronic structure and spectra of strongly correlated systems: The lda + *u* method. *J. Phys.: Condens. Matter* **9**, 767-808 (1997).

8 Dudarev, S. L., Botton, G. A., Savrasov, S. Y., Humphreys, C. J. & Sutton, A. P. Electron-energy-loss spectra and the structural stability of nickel oxide: An lsda + u study. *Phys. Rev. B* **57**, 1505-1509 (1998).

9 Vosko, S. H., Wilk, L. & Nusair, M. Accurate spin-dependent electron liquid correlation energies for local spin density calculations: A critical analysis. *Can. J. Phys.* **58**, 1200-1211 (1980).

10 Monkhorst, H. J. & Pack, J. D. Special points for brillouin-zone integrations. *Phys. Rev. B* **13**, 5188-5192 (1976).

11 Blochl, P. E., Jepsen, O. & Andersen, O. K. Improved tetrahedron method for brillouin-zone integrations. *Phys Rev B* **49**, 16223-16233 (1994).
